# Etiological Profile and Antimicrobial Patterns in Blood Culture Specimens in a Tertiary Care Setting

**DOI:** 10.7759/cureus.11000

**Published:** 2020-10-17

**Authors:** Asma Ejaz, Aneela Khawaja, Faiqa Arshad, Ambreen Tauseef, Rizwan Ullah, Ishtiaq Ahmad

**Affiliations:** 1 Pathology, Rahbar Medical & Dental College, Lahore, PAK; 2 Pathology, Gujranwala Medical College, Gujranwala, PAK; 3 Physiology, CMH Lahore Medical College and Institute of Dentistry, Lahore, PAK; 4 Community Medicine, Rahbar Medical & Dental College, Lahore, PAK; 5 Microbiology, Rahbar Medical & Dental College, Lahore, PAK

**Keywords:** blood stream infection, gram-positive cocci., antimicrobial resistance, gram-negative rods

## Abstract

Introduction

Universally, blood stream infections are linked with increasing morbidity and mortality. Timely diagnosis for identification of bacterial etiology, their susceptibility pattern and choice of empiric treatment plays a vital role in management.

Objective

To reveal the etiological profile and antibiotic sensitivity in blood culture specimens in a tertiary care setting.

Methods

This descriptive study was carried out in pathology laboratory of a tertiary care hospital from August 2016 to July 2019. All the 750 blood culture bottles were processed and isolates were recognized by morphological appearance on recommended media, gram stain, and different biochemical tests using Analytic Profile Index. Antibiotic sensitivity was implemented by modified disc diffusion method as per Clinical and Laboratory Standards Institute (CLSI) principles (2019).

Results

Out of 750 blood samples, 212 (28.26%) were culture positive. The percentage of gram-negative bacilli (n = 105) and gram-positive cocci (n = 104) was almost same (49.52%), while candida spp. was recovered from three (1.41%) isolates. The identified gram-negative bacteria were E. coli and Acinetobacter baumannii each (19.04%), Klebsiella pneumoniae and Pseudomonas aeruginosa each (16.19%), Enterobacter cloaca (11.42%), Salmonella typhi (8.57%), Burkholderia cepacia (1.90%), and Raoultella terrigena (7.61%). Among gram-positive isolates, coagulase-negative staphylococci (79.80%), Staphylococcus aureus (6.73%), Enterococcus spp. (11.53%) and Streptococcus spp. (1.92%) were recovered. Colistin, imipenem, meropenem, and amikacin were most successful against gram-negative rods. The sensitivity to vancomycin, teicoplanin and linezolid was 100%, for gram positive organisms. Methicillin resistance was present in 84.4% Staphylococcal isolates.

Conclusion

Local data showing changing etiological pattern and antibiogram of isolated pathogens, along with adequate infection prevention and control measures can be useful to improve patient care, in terms of hospital stay, duration of medication and treatment cost.

## Introduction

Septicemia and bacteremia are major contributors of hospitalization and mortality among all age groups. The factors through which bacteremia further progresses towards septicemia are overutilization of broad-spectrum antibiotics leading to colonization following antibiotic therapy, development of resistance and or continuation of resistant infection with lethal consequences [1]. This emerging resistance in bacterial pathogenesis is associated with deficient/poor contagion limitation procedures and lack of effective antimicrobial stewardship in healthcare centers, which end up in challenging outcomes globally [2].

Bacteremia is the presence and effective multiplication of pathogens in the circulatory system of a person with or without showing clinical features of sepsis. Most commonly isolated gram-positive organisms are staphylococci, streptococci and enterococci. Among gram-negative rods, E. coli,Klebsiella pneumoniae,Enterobacter cloaca, Salmonella typhi, Pseudomonas aeruginosa, and Acinetobacter baumannii, are often isolated [3]. Burkholderia cepacia has appeared as opportunistic multidrug resistant gram-negative non-lactose fermenter in immunocompromised subjects of all age groups. It has tendency to persist in antiseptic solutions as well as in hospital surroundings [4]. Raoultella terrigena, a gram-negative rod, is abundantly present in natural environment, water bodies and ecosystem. However, it is rarely associated with clinical infections and data from less developed countries is limited but special attention has to be given to the isolated resistant strains [5].

Culturing pathogenic organisms from hematogenous samples is a time-tested method to confirm bacteremia [6]. Empiric administration of antimicrobials according to the sensitivity patterns can reduce the development of multidrug resistance as well as overuse and misuse of antibiotics. Treatment options become limited, which can further complicate patient management [7].

This study was designed with an objective to identify the etiological profile and antimicrobial patterns in blood culture specimens in a tertiary care setting.

## Materials and methods

This study was done in microbiology laboratory of a tertiary care setting from August 2016 to July 2019. All blood culture samples collected from hospital admitted patients were included and repeat samples of same patient were excluded.

Blood samples of clinically suspected patients of septicemia collected before the administration of antibiotics in tryptic soya broth culture bottles. The culture bottles were incubated at recommended aerobic conditions. Next day broth culture was observed for any sign of bacterial growth. On day 2, first subculture from broth bottle was done on selective and differential media plates and incubated at 37°C. Smears made for Gram staining. The subculture plates were inoculated at day 4 and 7 as second and third subculture when signs of positivity appear. The negative subculture bottles were incubated for a period of seven days.

Preliminary identification was done by Gram staining, and bench tests, oxidase test and motility test by hanging drop method. Depending on Gram staining, and catalase reactions, gram-positive cocci (GPC) were divided into either staphylococci or streptococci/enterococci. The species differentiation of all the GPC was done by coagulase and DNAase tests, sensitivity to Novobiocin (5 µg) disc, and hemolytic reactions on blood agar. Grouping of beta hemolytic streptococci was done by Latex Kit (Pro-Lab Diagnostics, Merseyside, UK). Bile Esculin Agar (Oxoid CM0888) was used for identification of Group D streptococci. The gram-negative bacilli were distinguished by Analytical Profile Index (API 20E Biomerieux, France). Antibiotic sensitivity was identified by disk diffusion method following Clinical and Laboratory Standards Institute (CLSI) criteria (CLSI, 2019) [7]. The data was statistically evaluated with SPSS version 22.0 (IBM Corp., Armonk, NY). P-value ≤ 0.05 was considered significant.

## Results

Among 750 blood samples collected and processed in the laboratory, 212 (28.26%) were culture positive. Gram-negative rods (GNR) (n = 105) 50%, gram-positive cocci (GPC) (n = 104) 49%, and three isolates of Candida spp. (01%) were isolated from 212 positive blood culture samples. Gender wise distribution was almost equal i.e., male (n = 111) to female (n = 101) ratio was 52.35% and 47.64%, respectively. According to age, 115 samples belonged to less than 1 year age (54.2%), 67 among more than 20 years (31.6%), while 17 from less than 10 years (6%) and 13 from less than 20 years (8%). Among GNR, Escherichia coli and Acinetobacter spp. 20 each (19.04%), Klebsiella spp. and Pseudomonas spp. 17 each (16.19%), 12 Enterobacter spp. (11.42%), nine Salmonella spp. (8.57%), eight Raoultella terrigena (7.61%) and two Burkholderia cepacia (1.90%); among GPC, 83 Coagulase-negative staphylococci (CoNS) (79.80%), 12 Enterococcus spp. (11.53%), seven Staphylococcus aureus (6.73%) and two Streptococcus spp. (1.92%) were isolated (Table 1).

**Table 1:** Resistance pattern of isolated Gram-negative rods to different antibiotics (n = 105) 1- ampicillin 10µg, 2- amoxycillin-clavulanic acid 20/10µg, 3- piperacillin 100µg, 4- gentamicin 10µg, 5- ciprofloxacin 5µg, 6- cefuroxime 30µg, 7- cefotaxime 30µg, 8- ceftriaxone 30µg, 9- ceftazidime 30µg, 10- trimethoprim-sulfamethoxazole 25µg, 11- piperacillin-tazobactam 100/10µg, 12- imipenem 10µg, 13- meropenem 10µg, 14- amikacin 30µg.

Antibiotics	E. coli n = 20 (19.04%)	Kleb. spp. n = 17 (16.19%)	Enterobacter spp. n = 12 (11.42%)	Pseudomonas spp. n = 17 (16.19%)	Acinetobacter spp. n = 20 (19.04%)
AMP^1^	17 (85)	6 (35.29)	11 (91.66)	-	20 (100)
AMC^2^	14 (70)	5 (29.41)	9 (75)	-	20 (100)
PRL^3^	-	-	-	10 (58.82)	
CN^4^	10 (50)	4 (23.52)	5 (41.66)	7 (41.17)	9 (45)
CIP^5^	15 (75)	6 (35.29)	5 (41.66)	4 (23.52)	11 (55)
CXM^6^	13 (65)	3 (17.64)	8 (66.66)	14 (82.35)	20 (100)
CTX^7^	13 (65)	4 (23.52)	7 (58.33)	12 (70.58)	20 (100)
CRO^8^	13 (65)	0 (-)	7 (58.33)	11 (64.70)	20 (100)
CAZ^9^	-	-	-	13 (76.47)	15 (75)
SXT^10^	17 (85)	0 (-)	9 (75)	15 (88.23)	18 (90)
TZP^11^	11 (55)	5 (29.41)	6 (50)	7 (41.17)	17 (85)
IMP^12^	1 (05)	6 (35.29)	0 (-)	3 (17.64)	10 (50)
MEM^13^	0 (-)	5 (29.41)	7 (58.33)	6 (35.29)	14 (70)
AK^14^	8 (40)	3 (17.64)	8 (66.66)	5 (29.41)	7 (35)

The resistance trend of Gram-positive cocci (n = 104) is shown in Table 2. Sensitivity to vancomycin, teicoplanin and linezolid of all isolated GPCs was 100%. The isolated 12 Enterococcus spp. (11.53%) were 100% resistant to clindamycin and erythromycin, while resistance to ciprofloxacin and penicillin was 75% and 50%, respectively. Two isolates of Streptococcus spp. (1.92%), were 100% sensitive to the antibiotics tested according to CLSI, except one isolate was resistant (50%) to erythromycin, while, the other isolate showed resistance (50%) to ciprofloxacin (Table 2).

**Table 2:** Resistance trend Gram-positive cocci to antibiotics (n = 104) FOX-cefoxitin 30µg, DA-clindamycin 2µg, E-erythromycin 15µg, FA-fusidic acid 10µg

Antibiotics	CoNS n = 83 (%)	S. aureus n = 7 (%)
AMP^1^	83 (100)	6 (85.71)
AMC^2^	71 (85.54)	5 (71.42)
CN^4^	61 (73.49)	4 (57.14)
CIP^5^	70 (84.33)	6 (85.71)
CXM^6^	70 (84.33)	3 (42.85)
FOX	72 (86.74)	4 (57.14)
DA	64 (77.10)	5 (71.42)
E	77 (92.77)	6 (85.71)
FA	63 (75.90)	5 (71.42)
SXT^10^	72 (86.74)	3 (42.85)

## Discussion

Routine surveillance of blood-borne organisms is vital in scrutinizing the infectious pathogens about their sensitivity pattern in a specific region [8]. Knowledge of the baseline microbial resistance profile concerned with the hospital prevents irrational use of antibiotics in that hospital, thus helps progress a step forward in limiting spread of antibiotic resistance [9].

Our study demonstrated a blood culture positivity rate of 28.26% which is equivalent to other studies which reported a culture positivity rate of 28%, 32.7% and 25%, respectively [9-11]. Overall gram-negative isolates were more frequent (50%). This coincides with a local study by Qadeer et al. reporting 59% gram-negative organisms [12]. Some other studies from Kabul and India reported 51.7% and 51% gram-negative isolates, respectively [13,14]. In our study, the most common isolate is Staphylococcus epidermidis (79%). This is in accordance with a study in Lahore, reporting the highest percentage of Staphylococcus epidermidis (66.6%) among blood culture isolates [15]. Other international studies accounted for 41.2% and 57.3% respectively [16,17]. It is among the commonest reason of hospital acquired bloodstream infections and also the most frequent blood contaminant [3]. Single blood culture sample cannot evaluate truly positive bacteremia from false positive results due to contamination of skin flora. Extended use of intravascular devices could be another likely explanation for the isolated CoNS and or other underlying co-morbidities [18].

Two international studies reported Enterobacteriaceae as major isolate among gram-negative rods, i.e., 79.6% and 67%, respectively [11,18]. In present study, E. coli and Acinetobacter baumannii were isolated (19% each) followed by Klebsiella spp. and Pseudomonas spp. (16.19% each) and Enterobacter spp. (11.42%). This is in accordance with a local study by Qureshi and Aziz reporting 16% E. coli [17]. International studies reported E. coli to be isolated 32% and 26%, respectively [15, 16]. Other studies have documented Acinetobacter spp. to be most common [3,13].

A study from Peshawar, has revealed 57.1% blood culture positivity rate in neonates, which coincides with our study (54% in 0-1 yrs age) [19].

In vitro sensitivity of vancomycin and linezolid to be 100% has been reported by other studies as well [13]. Local study by Tariq reported 51% methicillin-resistant Staphylococcus aureus (MRSA) isolates [13]. Gram-positive isolates showed moderate to high resistance (above 50%) to ciprofloxacin, gentamycin and amoxicillin-clavulanic acid [3,17]. Sensitivity to colistin (100%) and other antibiotics as revealed by studies in Kashmir, India and Pakistan is in accordance to our study [12-15].

## Conclusions

Both Gram‑positive and Gram‑negative bacteria were found to be responsible for blood stream infections, and mostly were multidrug-resistant (MDR). This emphasizes the urgent need for rational use of antibiotics, proper antibiotic policy, and implementation of infection control practices for the active treatment and inhibition of drug resistance.

## References

[1] Goldstein E (2019). Relation between prescribing of different antibiotics and rates of bacteremia/septicemia and associated mortality in the US and England (PREPRINT). bioRxiv.

[2] Nobandegani AS, Motamedifar M (2019). Antibiotic sensitivity profile of the bacterial isolates from the blood samples of the patients in different wards of a major referral hospital, Shiraz, Iran 2015-2016. Pharmacophore.

[3] Vidyasagar K, Venkatesha D (2019). Study of microbiological profile and antibiotic susceptibility of blood stream infections in tertiary care hospital. Int J Curr Microbiol App Sci.

[4] Bansal S, Sharma R, Jangir N (2019). Pattern of clinical manifestation and antibiotics sensitivity of Burkholderia Cepacia sepsis in Neonatal Intensive Care Unit of tertiary care centre of North India. Int J Contemp Pediatr.

[5] Gajdács M (2019). Epidemiology of Raoultella species in the context of human infections: a 10-year retrospective study in a tertiary-care hospital in Hungary. Trends Med.

[6] Lim S, Yeom JS, Joo EJ (2019). Trends in bloodstream infections and antimicrobial susceptibilities at a university hospital in Korea between 2007 and 2016. Lab Med Online.

[7] Clinical and Laboratory Standards Institute (2019). Performance Standards for Antimicrobial Susceptibility Testing, 29 Ed.. https://clsi.org/media/2663/m100ed29_sample.pdf.

[8] Shrestha S, Amatya R, Shrestha RK, Shrestha R (2014). Frequency of blood culture isolates and their antibiogram in a teaching hospital. J Nepal Med Assoc.

[9] Sharma R, Sharma R, Gupta S (2015). Bacteriological analysis of blood culture isolates with their antibiogram from a tertiary care hospital. Int J Pharm Sci Res.

[10] Kamga HLF, Njunda AL, Nde PF, Assob JCN, Nsagha DS, Weledji P (2011). Prevalence of septicaemia and antibiotic sensitivity pattern of bacterial isolates at the University Teaching Hospital, Yaounde, Cameroon. African J Clin Exp Microbiol.

[11] Nazir A, Sana I, Peerzada BY, Farooq T (2018). Study of prevalence and antimicrobial susceptibility pattern of blood culture isolates from a tertiary care hospital of North India. Int J Res Med Sci.

[12] Qadeer S, Javed I, Mushtaq S, Anwar MS (2017). Trends in etiology and antimicrobial patterns in neonatal sepsis. A descriptive study in a tertiary care hospital, Lahore. Pak J Pathol.

[13] Tariq M (2014). Bacteriologic profile and antibiogram of blood culture isolates from a children's Hospital in Kabul. J Coll Physicians Surg Pak.

[14] Gupta S, Kashyap B (2016). Bacteriological profile and antibiogram of blood culture isolates from a tertiary care hospital of North India. Trop J Med Res.

[15] Lu B, Shi L, Zhu F, Zhao H (2013). Clinical utility of the time-to-positivity/procalcitonin ratio to predict bloodstream infection due to coagulase negative staphylococci. Lab Med.

[16] Kajumbula H, Fujita AW, Mbabazi O (2018). Antimicrobial drug resistance in blood culture isolates at a tertiary hospital, Uganda. Emerging Infect Dis.

[17] Qureshi M, Aziz F (2011). Prevalence of microbial isolates in blood cultures and their antimicrobial susceptibility profiles. Biomedica.

[18] Ullah O, Khan A, Ambreen A, Ahmad I, Akhtar T, Gandapor AJ, Khan AM (2016). Antibiotic sensitivity pattern of bacterial isolates of neonatal septicemia in Peshawar, Pakistan. Arch Iran Med.

[19] Mia AR, Zerin T (2020). Antibiogram of blood culture isolates of patients from a hospital in Dhaka, Bangladesh. Matrix Sci Medica.

